# Integration of GWAS SNPs and tissue specific expression profiling reveal discrete eQTLs for human traits in blood and brain

**DOI:** 10.1016/j.nbd.2012.03.020

**Published:** 2012-07

**Authors:** Dena G. Hernandez, Mike A. Nalls, Matthew Moore, Sean Chong, Allissa Dillman, Daniah Trabzuni, J. Raphael Gibbs, Mina Ryten, Sampath Arepalli, Michael E. Weale, Alan B. Zonderman, Juan Troncoso, Richard O'Brien, Robert Walker, Colin Smith, Stefania Bandinelli, Bryan J. Traynor, John Hardy, Andrew B. Singleton, Mark R. Cookson

**Affiliations:** a Laboratory of Neurogenetics, National Institute on Aging, National Institutes of Health, Bethesda, MD, USA; b Department of Molecular Neuroscience, UCL Institute of Neurology, London, UK; c Department of Medical & Molecular Genetics, King's College London, UK; d Research Resources Branch, National Institute on Aging, National Institutes of Health, Bethesda, MD, USA; e Brain Resource Center, Johns Hopkins University, Baltimore, MD, USA; f Geriatric Unit, Azienda Sanitaria Firenze (ASF), Florence, Italy; g Department of Pathology, The University of Edinburgh, Wilkie Building, Teviot Place, Edinburgh, UK; h Department of Neuroscience, Karolinska Institute, 171 77 Stockholm, Sweden

**Keywords:** eQTL, GWAS, Brain, Blood

## Abstract

Genome-wide association studies have nominated many genetic variants for common human traits, including diseases, but in many cases the underlying biological reason for a trait association is unknown. Subsets of genetic polymorphisms show a statistical association with transcript expression levels, and have therefore been nominated as expression quantitative trait loci (eQTL). However, many tissue and cell types have specific gene expression patterns and so it is not clear how frequently eQTLs found in one tissue type will be replicated in others. In the present study we used two appropriately powered sample series to examine the genetic control of gene expression in blood and brain. We find that while many eQTLs associated with human traits are shared between these two tissues, there are also examples where blood and brain differ, either by restricted gene expression patterns in one tissue or because of differences in how genetic variants are associated with transcript levels. These observations suggest that design of eQTL mapping experiments should consider tissue of interest for the disease or other traits studied.

## Highlights

► We integrate GWAS SNPs and examine the genetic control of gene expression in blood and brain tissue. ► Many eQTLs associated with human traits are shared between the blood and the brain. ► A number of discrete, tissue specific eQTLs also exist in the blood or the brain. ► Functional studies in blood have a limited capacity to inform on regulatory variation in the brain. ► Design of eQTL mapping experiments should consider the tissue of interest for the phenotype studied.

## Introduction

Genome-wide association (GWA) studies have provided novel insights into human traits by identifying single nucleotide polymorphisms (SNPs) associated with disease, including type 1 diabetes, coronary artery disease, HIV-1 infection and type 2 diabetes (Fellay et al., 2007, Preuss et al., 2010, Scott et al., 2007, Sladek et al., 2007, Steinthorsdottir et al., 2007, Todd et al., 2007, Yang et al., 2010, Zeggini et al., 2008), or other phenotypes. Because GWAS identify loci rather than functional variants, most GWAS have provided limited insights into underlying mechanisms (Hindorff et al., 2009). Therefore, annotating the possible functional effects of genetic risk variants is important in understanding genomic data.

Mapping of expression quantitative trait loci (eQTL) is one way to demonstrate that a risk variant within a locus has a functional effect on gene expression (Cheung et al., 2005, Morley et al., 2004, Myers et al., 2007, Stranger et al., 2007). eQTL analysis is performed by examining the association of each SNP with expression of mRNA transcripts. In general, eQTL effects are stronger for SNPs and transcripts that are physically close to each other (Gibbs et al., 2010). Trait associated SNPs from GWAS have been proposed to be more likely associated with expression differences than other SNPs (Nicolae et al., 2010). Such studies have generally been performed with transformed cell lines but eQTLs can also be identified in liver (Schadt et al., 2008), kidney (Wheeler et al., 2009), cell lines from asthma patients (Dixon et al., 2007, Moffatt et al., 2007) blood (Nalls et al., 2011a), subcutaneous adipose tissue (Emilsson et al., 2008) and brain (Gibbs et al., 2010, Heinzen et al., 2008, Liu et al., 2010a, Myers et al., 2007, Webster et al., 2009). For at least some loci, eQTLs are found consistently in both transformed cells and in primary tissues (Bullaughey et al., 2009). Overall, this data might suggest that functional annotation of GWAS loci can be performed in any convenient tissue.

Studying brain tissue is particularly challenging because these tissue samples have to be collected *post-mortem* and there is a high degree of cellular heterogeneity. Although some eQTLs have been nominated for brain diseases, such as MAPT in Parkinson's disease (PD) and progressive supranuclear palsy (PSP) (Hoglinger et al., 2011, Nalls et al., 2011b, Tobin et al., 2008, Vandrovcova et al., 2010), many nominated loci for brain phenotypes are not functionally annotated.

To explore the tissue specificity of eQTLs, we analyzed expression in brain and blood using SNPs abstracted from the NHGRI catalog of GWAS. We specifically wanted to address whether it is necessary to examine brain tissue to detect eQTLs for brain traits, including neurological diseases and psychiatric events, or whether the same information could be obtained from a more accessible tissue such as blood. We find that while many eQTLs are shared between blood and brain, there are specific instances, not always simply related to tissue specific gene expression levels, where the tissue studied limits detection of eQTLs.

## Materials and methods

### Samples

Fresh, frozen tissue samples from the frontal lobe of the cerebral cortex and from the cerebellum were obtained from neurologically normal Caucasian subjects. Genomic DNA was extracted using phenol–chloroform and RNA using Trizol from subdissected samples (100–200 mg). Peripheral blood specimens were collected using PAXgene tubes. RNA was extracted from peripheral blood samples using the PAXgene Blood mRNA kit (Qiagen, Crawley, UK) according to the manufacturer's instructions.

### Genotyping and imputation

Genotyping was performed using the Illumina Infinium HumanHap550 v3, Human610-Quad v1 or Human660W-Quad v1 Infinium Beadchip and common SNPs across all platforms were identified for each sample. SNPs were excluded if they showed < 95% genotyping success rate per SNP, minor allele frequency (MAF) < 0.01 or Hardy–Weinberg equilibrium (HWE) p-value < 1E − 7. Quality control was carried out using PLINK v1.07 for each cohort separately prior to imputation and was determined by comparing the subjects reported gender with the genotypic gender determined using PLINK's check sex algorithm.

Ethnicity and cryptic relatedness was determined using Identity-by-State (IBS) clustering and multidimensional scaling analyses within PLINK using genotypes that had been merged with data from HapMap Phase III, ASW, TSI, CEU, JPT, CHB and YRI populations [http://hapmap.ncbi.nlm.nih.gov/]. The subset of SNPs used was shared across studies, using only common SNPs that are not correlated within a 50 SNP sliding window at an r^2^ > 0.20, with each window overlapping by 5 SNPs. Samples were clustered using multidimensional scaling, removing outliers > 3 standard deviations from the mean component vector estimates for C1 or C2 for the combined CEU and TSI samples. Cryptically related samples were excluded after pairwise identical by descent estimates were calculated, excluding any samples sharing greater than a 0.15 proportion of alleles.

Markov Chain based haplotyper (MACH 1.0.16) was used to impute non-assayed genotypes for blood and brain datasets independently using the June 2010 release of the 1000 Genomes Project build-36 reference panel, using default settings for MACH. Imputed SNPs were excluded from the analysis if their minor allele frequency (MAF) was < 0.01 and if their r^2^ was < 0.3.

### GWAS SNPs

Trait and disease associated SNPs were extracted from the NHGRI catalog of published GWAS at http://www.genome.gov/gwastudies/ on July 30th 2011. Analyses were restricted to the following criteria: discovery p-value < 5E − 08, initial sample size > 1000 (or 1000 cases in binomial analyses), replication sample size > 500 (or 500 cases in binomial analyses), number of SNPs > 100,000, samples of European ancestry and risk allele frequency of SNP(s) greater or equal to 0.01.

### Expression profiling

Expression profiling was performed largely as previously described (Gibbs et al., 2010). RNA was biotinylated and amplified using the Illumina® TotalPrep-96 RNA Amplification Kit and directly hybridized onto HumanHT-12_v3 Expression BeadChips. Where possible, the same RNA samples were used from our previous study that used HumanRef8 Expression BeadChips. Raw intensity values for each probe were normalized using cubic spline in BeadStudio (Illumina) then log2 transformed. Individual probes were included in analysis if they were detected (P < 0.01) in more than 95% of samples in the series.

To define probes within +/− 1 MB of SNPs, probes were re-annotated using ReMOAT (http://www.compbio.group.cam.ac.uk/Resources/Annotation/). Ambiguous probes that mapped to multiple positions, or were identified as having design problems in ReMOAT, were excluded from subsequent analyses. To remove potential bias resulting from polymorphisms, all probes that included an analyzed SNP within the 50mer probe were removed.

### Expression QTL analyses

Starting with 447 subjects in the brain series, after data normalization and quality control, the brain mRNA dataset included 399 samples and ~ 9000 mRNA probes that were detected in > 95% of all samples. The blood dataset started with 712 samples, of which 501 passed all our QC steps; 5094 mRNA probes were detected in > 95% of samples.

In each brain region, mRNA probes within 500 kb of the chromosomal location of each SNP were incorporated into linear regression modeling using MACH2QTLv1.08. Estimates of the association between the allelic dose of each SNP as a predictor of proximal gene expression levels were generated. These linear regression models were adjusted for biological covariates of age at death and gender, the first 2 component vectors from multidimensional scaling, as well as methodological covariates including post-mortem interval (PMI), tissue bank and hybridization batch. SNPs with fewer than 3 minor homozygotes detected (based on either genotyped SNPs or maximum likelihood genotypes from imputation) were excluded from analyses. A consensus set of results was extracted from the frontal cortex, cerebellum and blood eQTL datasets with identical overlapping combinations of GWAS SNPs and proximal *cis* mRNA probes.

Significant associations were determined within each tissue type using a 5% FDR adjustment for multiple testing. Proportions of tested associations were calculated per tissue based on this subset of the eQTL results, and were compared using simple chi-squared tests.

### Case studies of specific loci

Identical statistical models were utilized to test our ability to detect known-associated eQTLs in previously published reports in tissues not previously investigated in GWAS. Results for these loci were mined for all associations within each +/− 500 kb region around top SNPs within each locus from the published GWAS within each tissue.

## Results

### Power to detect eQTLs in large blood or brain datasets

Directly comparing expression datasets derived from brain and whole blood in human samples is difficult because brain samples are taken *post-mortem* whereas blood samples are routinely taken during life. Therefore, we used two large, well-powered series from different sets of individuals to maximize our ability to find eQTLs in each tissue type. For brain, we expanded our previous dataset (Gibbs et al., 2010) in frontal cortex and cerebellum and obtained whole blood from 712 individuals from the InCHIANTI study (Wood et al., 2011). For consistency, we used the same expression array platform (Illumina HT-12 beadchips containing 48,000 probes) for all samples. After quality control, the brain mRNA dataset included 399 samples with data at 9000 probes. The blood dataset included 501 samples containing expression data from 5094 probes. Following imputation and quality control, ~ 2.2 million SNPs were available for analysis in all sample sets.

**Fig. 1:**
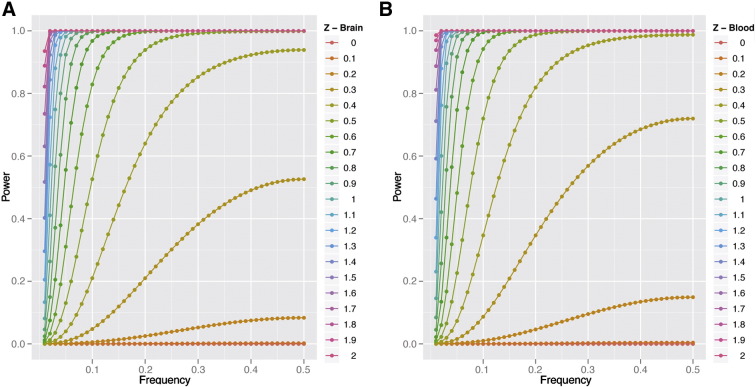
Power to detect eQTLs in brain and blood. Post-hoc power calculations were performed for sample sizes that we achieved after quality control in brain (A; 399 samples) or in blood (B; 501 samples). We estimated power (y axis) at a range of minor allele frequencies (x axis) for each sample series. Each colored line represents a different normalized effect size (Z) varying from 0.1 to 2.0 standard deviations of difference for each minor allele in an additive model. The steeper power curves for the blood series (B) indicate improved power over brain (A) to detect the same effect size, given the lower number of samples in the former series.

Because the final number of samples within the blood and brain groups differed, we performed *post-hoc* power calculations to compare ability to detect eQTLs (Fig. 1). Based on our previous work in brain (Gibbs et al., 2010), the strength of the association varies substantially for different eQTLs. Therefore, we estimated power over a range of minor allele frequencies and of effect sizes for the eQTLs, using Z as a measure of effect size standard deviations of difference for each minor allele under an additive model. As an example of power in the two datasets at a realistic pair of these parameters, the blood dataset had 98.8% power to detect eQTLs at an effect allele frequency of 0.2 and an additive effect size of Z = 0.5 whereas the brain dataset had 93.9% power to detect the same magnitude of effect. This analysis demonstrates that the difference in power in the two datasets is minimized as the fraction of true eQTL effect sizes rises. For eQTLs with moderate effect sizes (Z > 0.2) we were reasonably powered in both series; therefore, we proceeded to compare the ability to detect eQTLs in both datasets.

### Gene expression in blood versus brain in human populations

**Fig. 2:**
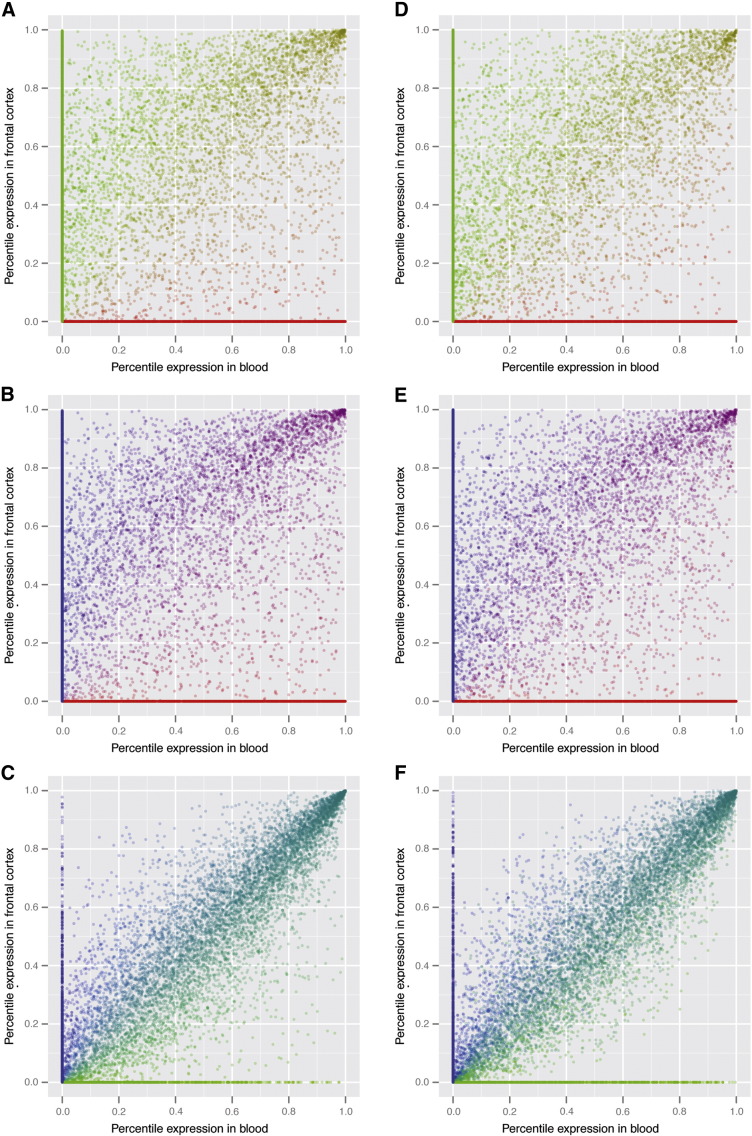
Comparative gene expression in blood and in brain. (A–C) Normalized gene expression values for each probe on the microarrays were converted to mean values across the population and ranked such that 1.0 is the highest expressed gene. Where genes were detected in < 95% of samples in the population, we set the percentile to 0. We plotted these to compare expression in blood versus frontal cortex (A) or cerebellum (B), or to compare frontal cortex and cerebellum (C). Each probe is color coded by the difference in rank between the pairs of tissue. (D–F) Similar plot but for percentile rank of the variance in expression across the population of samples for blood versus frontal cortex (D) or cerebellum (E), or frontal cortex and cerebellum (F).

It is expected that gene expression profiles would be divergent between blood and brain tissues but similar for two brain regions. To test this, we ranked as percentiles the normalized gene expression values averaged for all subjects, setting non-detected probes to zero. Gene expression values were shown to be highly divergent between blood and either frontal cortex or cerebellum tissue for a large number of genes that were only detected reliably in one tissue or the other (Figs. 2A,B). In contrast, gene expression was more similar between frontal cortex and cerebellum and there were fewer uniquely expressed genes (Fig. 2C). Analysis using percentile ranked variance rather than mean values for each probe yielded similar results (Figs. 2D–F), showing that mean expression and variance in expression were closer in the two brain regions than in blood.

### eQTL discovery for genes expressed in blood and brain

We next examined the relative ability of the three datasets to detect eQTLs from regions nominated in GWAS. We abstracted SNPs associated with human traits based on the NHGRI catalog of GWA, yielding1366 loci. Of these, 783 SNPs passed the criteria of having a replicated association with traits or diseases and being within 0.5 MB of the chromosomal position of a probe for gene expression. We chose the threshold of 0.5 MB based on previous data (Gibbs et al., 2010) where we saw the average distance between a SNP and significant eQTL was 121 Kb and > 90% of significant eQTLs were detected within 0.5 MB.

**Fig. 3:**
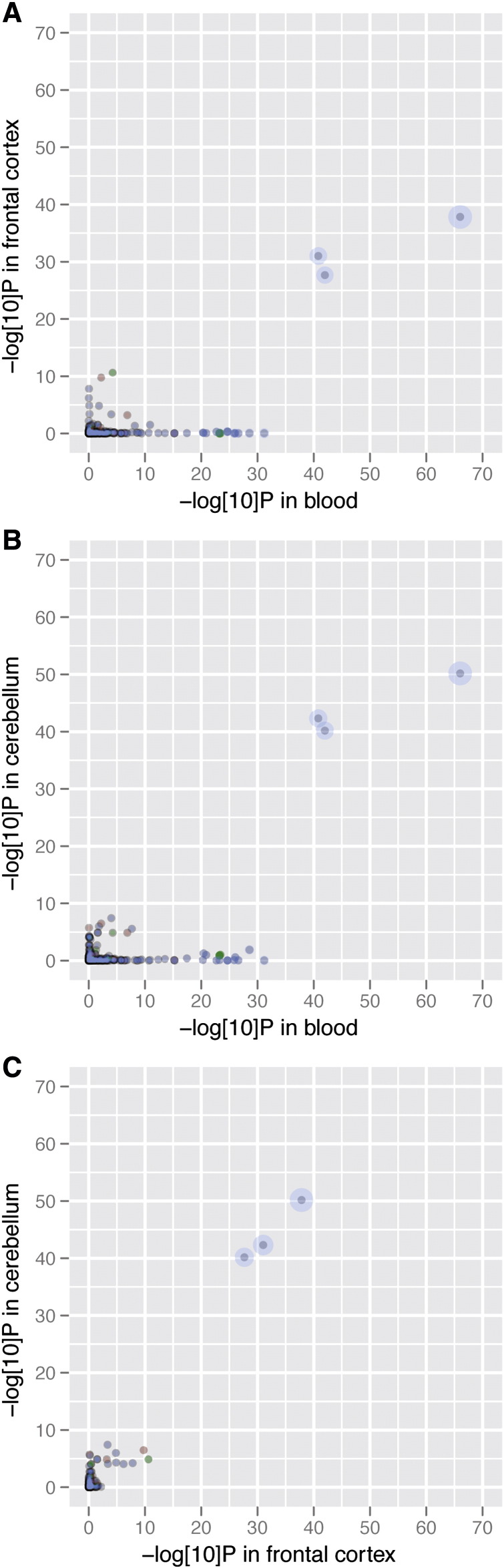
Similar and distinct SNP:probe associations in brain and blood. Each point shows comparisons of − log[10] of FDR corrected p values for identical SNP and probe combinations across all 3 tissues investigated, comparing blood with frontal cortex (A) or cerebellum (B) and frontal cortex to cerebellum (C). Size of points is scaled to the combined FDR corrected p values after − log[10] transformation. Points are colored by the associated phenotypes, where brain traits are shown in orange, blood traits in green and others in blue.

We manually annotated the traits studied in each GWAS as related to blood (176 SNPs), brain (61 SNPs) or other (546 SNPs) phenotypes (Supplementary file 1). For example, we annotated traits associated with neurological or psychiatric conditions as “brain” and markers of subtypes of blood cell markers as “blood”. We then used this list of SNPs to perform eQTL analysis. We first performed the eQTL analysis in a uniform way by only considering the subset of probes and SNPs detected in all tissue types, or 2929 SNP:probe pairs. This analysis identified eQTLs that were highly significant in all three tissues and additional eQTLs distinctly significant in either blood or brain tissues (Fig. 3). Of the shared eQTLs, three stood out as highly significant in all three tissues for three SNPs including a single mRNA probe, ILMN_1695585 that maps to the *RPS26* gene on chromosome 12q13.2, within 500 KB of three GWAS SNPs associated with Type 1 diabetes (False discovery rate (FDR) corrected *P* < 1.45 × 10^− 38^ for association with rs11171739 in the frontal cortex, *P* < 6.72 × 10^− 51^ in cerebellum and *P* < 9.46 × 10^− 67^ in blood) (Barrett et al., 2009, 2007; Cooper et al., 2008, Hakonarson et al., 2008, Todd et al., 2007). Additional significant SNP:probe pairs found in both datasets included SNPs associated with traits such as mean corpuscular volume (Ganesh et al., 2009) , smoking behavior (2010), eye color (Liu et al., 2010b), plasma levels of liver enzymes(Yuan et al., 2008) and inflammatory bowel disease (Kugathasan et al., 2008) (Supplementary Table 1). For fifteen SNP:probe associations that were significant in brain and blood, the direction of effect was consistent across all three tissues.

A divergent set of eQTLs were found in the blood dataset when compared with cerebellum and frontal cortex (Figs. 3A,B). Several of these eQTLs were for probe ILMN_1666206, which maps to the *GSDML* gene on Chr17q12. These correlations are linked with five separate GWA studies associating Type 1 diabetes (2007; Barrett et al., 2009, Cooper et al., 2008, Hakonarson et al., 2008, Todd et al., 2007), Crohn's disease (Barrett et al., 2008) and Ulcerative colitis (McGovern et al., 2010) to the same locus (Supplementary Table 1). Additionally, the ILMN_1666206 probe has been nominated as underlying an eQTL in studies of asthma and white blood cell traits associated with a pro-inflammatory state (Moffatt et al., 2007, Nalls et al., 2011a). The FDR corrected *P* value for the most significantly associated SNP, rs2290400, with this probe was 6.41 × 10^− 32^ in blood but 0.924 and 0.896 in the cerebellum and frontal cortex respectively.

**Fig. 4:**
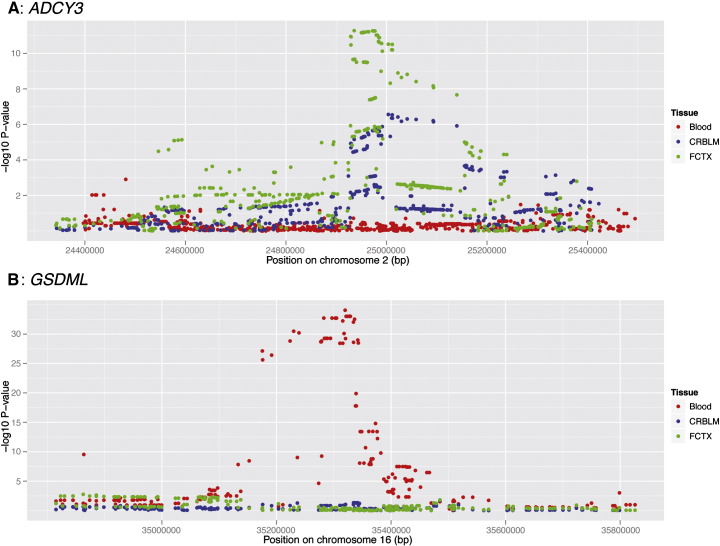
Blood and brain specific eQTLs in probes that are detected in all tissues. (A) Similar locus plot for ILMN_167893, which maps to *ADCY3* and reveals a highly significant signal in the brain samples but no significant p values in blood, despite adequate detection of the probe in all tissues. (B) Plot of SNPs along the Chr17 region that includes the *GSDML* and *ORMDL3* genes showing − log[10]P values for association of each SNP with expression of Illumina probe ILMN_1666206, which maps to the *GSDML* gene. Despite having significant detection in all three tissues, there was a strong signal for blood (red) but not in either of cerebellum (blue) or frontal cortex (green).

To examine this phenomenon further, we compared all SNPs within the *GSDML/ORMDL3* region to the expression of ILMN_1666206 in all tissues. Although expression was detected, we did not find significant associations with any SNPs in the brain, but found strong associations between probe expression and proximal SNPs in blood tissue (Fig. 4B). This locus therefore represents an example of a blood-specific eQTL.

Conversely, a subset of SNP:probe pairs reached significance in the brain samples but not in blood (Figs. 3A,B and Supplementary Table 1). For example, rs713586, which was nominated for association with body mass index (PMID: 20935630), was significantly associated with the expression of ILMN_1676893 in cerebellum (FDR corrected *P* = 6.09x10^− 5^) and frontal cortex (FDR corrected *P* = 1.53 × 10^− 8^) but showed no association in blood (FDR corrected *P* = 0.89). This probe maps to the adenylate cyclase gene *ADCY3* on chromosome 2 (Fig. 4A). Interestingly, variation in *ADCY3* has been nominated in a number of GWAS including for alcohol dependence (Edenberg et al., 2010) and major depression (Wray et al., 2010).

Overall this data suggests that while some eQTLs are consistent between tissues, there is a subset where a genetic effect on gene expression exists in one tissue context but not the other, despite probe detection in both instances.

### eQTL discovery for genes with expression restricted to blood or brain

Given that there were differences in gene expression between tissues (Fig. 2), we next analyzed eQTLs unique to each tissue by examining the association of GWAS SNPs with expression of probes detected in either blood or brain but not in both (Supplementary Table 2).

**Fig. 5:**
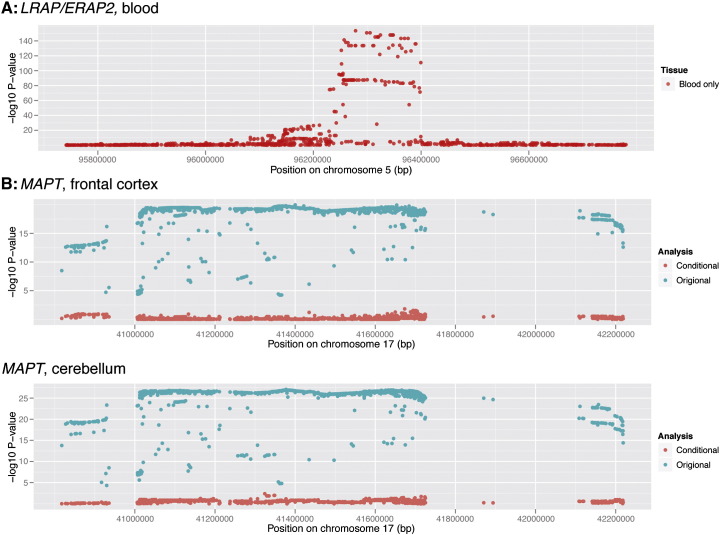
eQTLs in probes detected only in brain or blood. (A) SNPs along the region of Chr5 that contains the *LRAP* gene showing − log[10]P values for association of each SNP with expression of Illumina probe ILMN_1743143. (B) Plot of SNPs along the Chr17 region that includes the *MAPT* gene for ILMN_ 1710903 in frontal cortex (upper panel) or cerebellum (lower panel). For each tissue, we repeated the original eQTL analysis (green) but made the analysis conditional on a proxy SNP for the H1/H2 inversion haplotype (orange). The decrease in P values after conditioning on a proxy SNP suggests that most of the signal arises from the H1/H2 haplotype.

In blood, there was a highly significant (FDR corrected *P* = 1.27 × 10^− 131^) association between rs2549794 at a Crohn's disease locus (Franke et al., 2010) and expression of ILMN_1743145, which maps to the *LRAP/ERAP2* gene (Fig. 5A). Other associations measurable only in the blood datasets include rs2304130 and ILMN_2134224, and rs6120849 and ILMN_2402805. These SNPs were nominated as associated with measurements of total cholesterol and protein C respectively in plasma (Tang et al., 2010, Waterworth et al., 2010).

We also found a series of significant associations in brain and not blood. Several of these associations were probes on chromosome 12 associated with rs11171739 or rs1701704, two SNPs nominated for Type-I diabetes. Additional associations included a series of SNPs on chromosome 17. Previous studies have noted an effect of chromosome17 SNPs on expression of the *MAPT* gene that is associated with risk of PD and PSP (2011; Hoglinger et al., 2011). We therefore examined expression of ILM_1710903, which maps within the coding sequence of *MAPT*, with association of SNPs across Chr17 and saw robust signals in both the frontal cortex and cerebellum (Fig. 5B). This effect was driven by the H1/H2 haplotype across the *MAPT* locus as conditioning the analysis on a proxy SNP decreased the apparent eQTL signal.

Collectively, these results show that while some eQTLs are shared across tissues, there are examples where restricted expression levels in one tissue limit the ability to detect significant associations.

### Overall ability to detect eQTLs depends on tissue type and gene expression

We next compared the proportions of eQTLs found in the three sample series and considered whether these were associated with brain, blood or other phenotypes (Table 1). In the analysis restricted to probes detected in both blood and brain tissues, 125 eQTLs were found within 500 KB of any GWA SNP in blood, versus 40 eQTLs found in the brain dataset. Of these, 21 significant eQTLs were found in the blood dataset for blood traits, while 16 eQTLs were found in blood for brain traits. Six significant eQTLs were found in the brain, counting either cerebellum or frontal cortex, for brain traits and an additional six eQTLs were found in brain for blood traits out of 40 total significant associations. The proportions of blood and brain traits with detected eQTLs were similar in blood and brain samples (two tailed Z-test, *P* = 0.18, 1.0 respectively).

We performed independent analyses of probes that were only detected in one tissue as more probes were tested in brain (1877 in frontal cortex and 1853 in cerebellum) than in blood (413). There were 107 significant associations in cerebellum and 90 in frontal cortex compared to 21 in blood but we did not find over-representation of traits annotated as brain related in the brain datasets (Table 2).

## Discussion

We have performed an eQTL analysis using SNPs from the NHGRI catalog of GWAS in two tissue types, blood and brain (frontal cortex and cerebellum). The nominated SNPs are associated with a variety of human traits, including diseases, physiological markers such as blood cell numbers and continuous traits such as height. We specifically addressed whether it is necessary to examine brain tissue to detect eQTLs for brain phenotypes or whether the same information could be obtained from blood. We find that while many eQTLs are shared between blood and brain tissues, there are specific instances, not always simply related to gene expression levels, where the detection of eQTLs is limited by the tissue studied.

A small number of eQTLs are detectable in all three datasets tested. A proportion of these common eQTLs demonstrated strong effect sizes, such as SNPs associated with Type 1 diabetes on chromosome 12 (Barrett et al., 2009, Burton et al., 2007, Cooper et al., 2008, Hakonarson et al., 2008, Todd et al., 2007) or associated with smoking behavior on chromosome 19 (Furberg et al., 2010). We have therefore demonstrated that coincident eQTLs exist between blood and brain tissues and therefore discrete eQTLs are found in more than one human primary tissue as previously suggested (Bullaughey et al., 2009, Ding et al., 2010, Greenawalt et al., 2011).

There were eQTLs that could be detected only in one tissue type and in some cases these are due to differences in gene expression. This is true for genes such as *MAPT,* which encodes for the tau protein that is expressed largely in post-mitotic neurons. Therefore, there will be cases where, when interrogating GWAS data it will be important to examine the target tissue of interest, thus affirming the need to look at brain for studies related to neurological or psychiatric phenotypes.

Of greater interest is that we also found a subset of eQTLs that appear to be tissue specific, despite the probes being reliably detected in all samples series. It is possible that genetic variants can affect expression levels exclusively in a subset of tissues. For example, gene expression may be altered in a tissue- and timing-specific manner by *cis*-regulatory elements (Cooper et al., 2008). In this case, although multiple tissues may be permissive for expression, different *-cis* regulatory elements are being employed in each tissue and lead to quantitatively different expression levels. Understanding why there are examples where differences in expression do not explain eQTL detection in a simple way will be an important question for future studies.

One caveat to these studies is that direct comparison of datasets derived from separate tissue types with differential ascertainment methods is difficult. Specifically, the brain samples were taken from deceased subjects whereas blood samples were drawn in life. However, post-mortem interval has been shown not to be a major confound within brain expression data (Gibbs et al., 2010, Trabzuni et al., 2011) and we corrected for this and other known methodological variables in the statistical model. However, because the samples used here were from different individuals, we cannot exclude that we are detecting rare alleles and/or genetic variants on a background of common SNPs. As demonstrated by power analysis, the current dataset is not powered to directly detect rare alleles but has good power to detect relatively large eQTL effect sizes. Therefore, this analysis performs best for loci that are tagged by common variants and where the effect of the minor allele on expression is relatively large. It is also important to note that in the present study, we limited our analysis to transcripts within a relatively narrow (0.5 MB) window around each SNP. This is larger than the average distance between SNP and associated transcript of 121 Kb (Gibbs et al., 2010) but may inadvertently omit true eQTLs at larger distances while maintaining power. Larger series would be needed to expand the analyses to more distal effects.

Further dissection of such loci will likely require deep sequencing of the genome for many individuals and additional large-scale studies. One general limitation of hybridization based arrays is that detection of low expression genes is difficult, which may be overcome by RNA sequencing in the future. In addition to both of these technological developments, eQTL surveys such as the one presented here will need to be repeated as the number of SNPs nominated by GWAS studies increases. This is perhaps particularly true for brain related phenotypes. Although we did not find that there were significantly more eQTLs for brain phenotypes using brain expression data, the number of replicated GWAS ‘hits’ for neurological and psychiatric conditions is still quite small and we might expect the brain to be more sensitive as the number of replicated loci increases. We have not tested all possible SNPs in the current analysis to maintain power to detect significant associations, but such analyses could be performed on an ad hoc basis for nominated SNPs in future GWAS without the loss of power caused by testing the whole genome.

Overall, we demonstrate a number of clear and key examples where brain tissue is required for eQTL discovery. We conclude that functional studies in one tissue have the capacity to inform our understanding of regulatory variation in general, but that there are sufficient numbers of counter-examples to suggest that for neurological and psychiatric traits we should continue to examine gene expression in the brain.

### Datasets

Datasets have been submitted to GEO: accession # GSE36192.

The following are the supplementary materials related to this article.

Supplementary material

Supplemental Table 1 Significant genotype/expression associations for probes that were detected in all tissues.

Supplemental Table 2 Significant genotype/expression associations for probes that were detected either in blood or brain.

## Figures and Tables

**Table 1:** Counts of SNPs and SNP:probe pairs tested and significant associations per tissue and trait for probes that were detected in all tissues.

	Counts of all SNPs and probes used and their annotations as blood vs brain			
All GWAS	Blood	Brain	Other
SNPs	783	176 (22.5%)	61 (7.8%)	546 (69.7%)
SNP:probe pairs	2929	683 (23.3%)	227 (7.6%)	2019 (68.9%)

	Count (% of all) of significant associations within 500 kb of SNP			
Blood	125	21 (16.8%)	16 (12.8%)	88 (70.4%)
Cerebellum	33	5 (15.15%)	5 (15.15%)	23 (69.7%)
Frontal cortex	21	3 (14.3%)	2 (9.5%)	16 (76.2%)

**Table 2:** Counts of SNPs and SNP:probe pairs tested and significant associations per tissue and trait for probes that were detected in either blood or in brain.

				Count (% of all) of significant associations within 500 kb of SNP			
Tissue	SNPs	Probes	Associations tested	All GWAS	Blood	Brain	Other
Blood	648	413	658	21	5 (23.8%)	2 (9.5%)	14 (66.7%)
Cerebellum	943	1853	3924	107	21 (19.6%)	13 (12.1%)	73 (68.2%)
Frontal cortex	978	1877	3968	90	21 (23.3%)	7 (7.8%)	62 (68.9%)
