# Supplementary material for: Integration of GWAS SNPs and tissue specific expression profiling reveal discrete eQTLs for human traits in blood and brain
Source: Neurobiol Dis. 2012 Jul;47(1):20–8. doi: 10.1016/j.nbd.2012.03.020 (PMC3358430; doi:10.1016/j.nbd.2012.03.020)
Supplement: Supplemental Table 1 — Significant genotype/expression associations for probes that were detected in all tissues. [file mmc2.doc]

Supplemental Table 2. Significant genotype/expression associations for probes that were detected either blood or brain

| SNP | GWAS | Type | Probe | Gene | Chr | FDR adjusted *P* |
| --- | --- | --- | --- | --- | --- | --- |
| (a) Associations for probes detected in blood, *P* values for blood | | | | | | |
| rs2549794 | Crohn's disease | Other | ILMN_1743145 | ERAP2 | 5 | 1.27E-131 |
| rs2304130 | Triglycerides | Blood | ILMN_2134224 | ATP13A1 | 19 | 1.20E-63 |
| rs6120849 | Plasma levels of Protein C | Blood | ILMN_2402805 | TRPC4AP | 20 | 6.99E-21 |
| rs2058660 | Crohn's disease | Other | ILMN_1721762 | IL18RAP | 2 | 1.39E-20 |
| rs917997 | Celiac disease | Other | ILMN_1721762 | IL18RAP | 2 | 1.39E-20 |
| rs6904029 | Vitiligo | Other | ILMN_2203950 | HLA-A | 6 | 1.45E-15 |
| rs30187 | Ankylosing spondylitis | Other | ILMN_1743145 | ERAP2 | 5 | 1.85E-14 |
| rs660895 | Rheumatoid arthritis | Other | ILMN_1808405 | XM_001719804^1^ | 6 | 6.91E-09 |
| rs7255045 | Mean corpuscular volume | Blood | ILMN_1796245 | 2 | 19 | 3.07E-07 |
| rs131794 | Mean corpuscular volume | Blood | ILMN_1690939 | TYMP | 22 | 2.05E-05 |
| (b) Associations for probes detected in brain, *P* values for cerebellum | | | | | | |
| rs11171739 | Type 1 diabetes | Other | ILMN_1726647 | RPS26^3^ | 12 | 1.50E-49 |
| rs11171739 | Type 1 diabetes | Other | ILMN_2209027 | RPS26^3^ | 12 | 1.17E-48 |
| rs11171739 | Type 1 diabetes | Other | ILMN_2310703 | RPS26^4^ | 12 | 1.86E-47 |
| rs11171739 | Type 1 diabetes | Other | ILMN_1678522 | XM_930344^5^ | 12 | 5.32E-44 |
| rs1701704 | Type 1 diabetes | Other | ILMN_1726647 | RPS26^3^ | 12 | 5.34E-43 |
| rs11171739 | Type 1 diabetes | Other | ILMN_1750636 | RPS26^4^ | 12 | 1.35E-42 |
| rs8070723 | Parkinson's disease | Brain | ILMN_2393693 | XR_037772^5^ | 17 | 4.37E-41 |
| rs1701704 | Type 1 diabetes | Other | ILMN_2310703 | NR_002225^4^ | 12 | 1.13E-40 |
| rs1701704 | Type 1 diabetes | Other | ILMN_2209027 | RPS26^3^ | 12 | 3.90E-40 |
| rs2292239 | Type 1 diabetes | Other | ILMN_1726647 | RPS26^3^ | 12 | 5.59E-40 |
| rs2942168 | Parkinson's disease | Brain | ILMN_2393693 | XR_037772^5^ | 17 | 1.09E-39 |
| rs393152 | Parkinson's disease | Brain | ILMN_2393693 | XR_037772^5^ | 17 | 1.09E-39 |
| rs2292239 | Type 1 diabetes | Other | ILMN_2310703 | NR_002225 | 12 | 2.11E-38 |
| rs2292239 | Type 1 diabetes | Other | ILMN_2209027 | RPS26^3^ | 12 | 2.77E-38 |
| rs1701704 | Type 1 diabetes | Other | ILMN_1750636 | NR_002225^4^ | 12 | 4.65E-38 |
| rs1701704 | Type 1 diabetes | Other | ILMN_1678522 | XM_930344^5^ | 12 | 9.07E-38 |
| rs2292239 | Type 1 diabetes | Other | ILMN_1750636 | NR_002225^4^ | 12 | 1.88E-35 |
| rs2292239 | Type 1 diabetes | Other | ILMN_1678522 | XM_930344^5^ | 12 | 3.73E-35 |
| rs8070723 | Parkinson's disease | Brain | ILMN_1710903 | MAPT | 17 | 1.51E-29 |
| rs10781499 | Ulcerative colitis | Other | ILMN_1811301 | INPP5E | 9 | 1.95E-29 |
| rs4077515 | Crohns disease | Other | ILMN_1811301 | INPP5E | 9 | 1.95E-29 |
| rs2942168 | Parkinson's disease | Brain | ILMN_1710903 | MAPT | 17 | 2.81E-28 |
| rs393152 | Parkinson's disease | Brain | ILMN_1710903 | MAPT | 17 | 2.91E-28 |

^1^ HLA class II histocompatibility antigen, DQ alpha 1 chain-like

^2^ probe failed QC on REMOAT

^3^ Two probes may map to RPS26 or a pseudogene

^4^ NR_002225 may map to the RPS26 pseudogene cluster on chr12

^5^Hypothetical proteins, unclear if transcribed
