# Supplementary material for: Integration of GWAS SNPs and tissue specific expression profiling reveal discrete eQTLs for human traits in blood and brain
Source: Neurobiol Dis. 2012 Jul;47(1):20–8. doi: 10.1016/j.nbd.2012.03.020 (PMC3358430; doi:10.1016/j.nbd.2012.03.020)
Supplement: Supplemental Table 2 — Significant genotype/expression associations for probes that were detected either in blood or brain. [file mmc3.doc]

Supplemental Table 1. Significant genotype/expression associations for probes that were detected in all tissues

|  |  |  |  |  |  | FDR adjusted *P* value | | |
| --- | --- | --- | --- | --- | --- | --- | --- | --- |
| SNP | GWAS | Type | Probe | Gene | Chr | Blood | Cerebellum | Frontal Cortex |
| (a) SNP:Probe pairs significant in all three tissues | | | | | | | | |
| rs11171739 | Type 1 diabetes | Other | ILMN_1695585 | RPS26^1^ | 12 | 9.46E-67 | 6.72E-51 | 1.45E-38 |
| rs1701704 | Type 1 diabetes | Other | ILMN_1695585 | RPS26 | 12 | 1.57E-41 | 4.86E-43 | 9.69E-32 |
| rs2292239 | Type 1 diabetes | Other | ILMN_1695585 | RPS26 | 12 | 1.04E-42 | 6.73E-41 | 2.14E-28 |
| rs3733829 | Smoking behavior | Brain | ILMN_2109994 | RAB4B | 19 | 5.44E-05 | 1.36E-05 | 2.48E-11 |
| rs11597390 | Plasma levels of liver enzymes | Blood | ILMN_1651886 | CWF19L1 | 10 | 0.006086 | 3.33E-07 | 1.70E-10 |
| rs7665090 | Primary biliary cirrhosis | Other | ILMN_1800733 | MANBA | 4 | 0.01492 | 1.04E-06 | 1.47E-05 |
| rs1003719 | Eye color | Other | ILMN_1764871 | PIGP | 21 | 9.65E-05 | 3.79E-08 | 0.0004479 |
| rs131794 | Mean corpuscular volume | Blood | ILMN_2109708 | TYMP | 22 | 1.38E-07 | 1.35E-05 | 0.0006268 |
| rs2315008 | Inflammatory bowel disease | Other | ILMN_1794643 | ZGPAT | 20 | 0.02410 | 1.28E-05 | 0.03064 |
| rs4809330 | Crohn’s disease | Other | ILMN_1794643 | ZGPAT | 20 | 0.02410 | 1.35E-05 | 0.03064 |
| (b) SNP:Probe pairs significant in blood but not in brain | | | | | | | | |
| rs2290400 | Type 1 diabetes | Other | ILMN_1666206 | GSDMB | 17 | 6.41E-32 | 0.9239 | 0.8960 |
| rs2872507 | Crohn's disease | Other | ILMN_1666206 | GSDMB | 17 | 2.54E-27 | 0.8789 | 0.9300 |
| rs2290400 | Type 1 diabetes | Other | ILMN_2347193 | GSDMB | 17 | 8.97E-27 | 0.2658 | 0.8749 |
| rs2305480 | Ulcerative colitis | Other | ILMN_1666206 | GSDMB | 17 | 1.34E-26 | 0.8138 | 0.9479 |
| rs7359397 | Body mass index | Other | ILMN_1738369 | TUFM | 16 | 2.13E-25 | 0.9303 | 0.4791 |
| rs7498665 | Body mass index | Other | ILMN_1738369 | TUFM | 16 | 2.13E-25 | 0.9303 | 0.4791 |
| rs8070723 | Parkinson’s disease | Brain | ILMN_1784428 | NR_026680 | 17 | 3.91E-24 | 0.09533 | 0.9419 |
| rs1728785 | Ulcerative colitis | Other | ILMN_1684628 | ZFP90 | 16 | 5.59E-24 | 0.8133 | 0.7402 |
| rs2942168 | Parkinson’s disease | Brain | ILMN_1784428 | NR_026680 | 17 | 5.70E-24 | 0.1265 | 0.9550 |
| rs393152 | Error: Reference source not found | Brain | ILMN_1784428 | NR_026680 | 17 | 5.70E-24 | 0.1265 | 0.9550 |
| (c) SNP:Probe pairs significant in brain but not in blood | | | | | | | | |
| rs2665838 | Height | Other | ILMN_1695983 | DDX42 | 17 | 0.7257 | 8.52E-05 | 0.0003857 |
| rs46522 | Coronary heart disease | Other | ILMN_1692168 | UBE2Z | 17 | 0.7907 | 4.87E-05 | 1.32E-05 |
| rs713586 | Body mass index | Other | ILMN_1676893 | ADCY3 | 2 | 0.8899 | 6.09E-05 | 1.53E-08 |
| rs4665736 | Height | Other | ILMN_1676893 | ADCY3 | 2 | 0.9555 | 8.89E-05 | 6.39E-07 |
| rs185819 | Height | Other | ILMN_2390416 | BAT3 | 6 | 0.9836 | 0.8327 | 0.006185 |
| rs8170 | Breast cancer | Other | ILMN_1783681 | MRPL34 | 19 | 0.1660 | 0.5294 | 0.02051 |
| rs1165205 | Serum urate | Blood | ILMN_1651496 | HIST1H2BD | 6 | 0.3340 | 0.1894 | 0.03064 |
| rs2814993 | Height | Other | ILMN_2086064 | SNRPC | 6 | 0.6083 | 0.9417 | 0.03866 |
| rs2523393 | Multiple sclerosis | Brain | ILMN_1747598 | PPP1R11 | 6 | 0.2628 | 0.3808 | 0.04502 |
| rs11239550 | Mean corpuscular volume | Blood | ILMN_1680996 | ALOX5 | 10 | 0.8881 | 1.84E-06 | 0.6866 |
| rs2942168 | Parkinson’s Disease | Brain | ILMN_1709549 | PLEKHM1 | 17 | 0.6502 | 8.89E-05 | 0.3854 |
| rs393152 | Parkinson’s Disease | Brain | ILMN_1709549 | PLEKHM1 | 17 | 0.6502 | 8.99E-05 | 0.3854 |
| rs734999 | Ulcerative colitis | Other | ILMN_1697409 | TNFRSF14 | 1 | 0.4946 | 0.000205 | 0.7818 |
| rs2294008 | Bladder cancer | Other | ILMN_1777740 | C8orf55 | 8 | 0.9451 | 0.0018497 | 0.3174 |
| rs17318596 | Height | Other | ILMN_1659725 | EXOSC5 | 19 | 0.7525 | 0.001932 | 0.4004 |
| rs2297441 | Ulcerative colitis | Other | ILMN_1794643 | ZGPAT | 20 | 0.7343 | 0.001969 | 0.6513 |
| rs2251219 | Major mood disorders | Brain | ILMN_1657810 | PPM1M | 3 | 0.5368 | 0.01016 | 0.4911 |
| rs511154 | Fibrinogen | Blood | ILMN_1761010 | PCCB | 3 | 0.7159 | 0.01016 | 0.2193 |
| rs6010620 | Glioma | Other | ILMN_1794643 | ZGPAT | 20 | 0.8378 | 0.01098 | 0.6252 |
| rs2749097 | Alcohol consumption | Brain | ILMN_1800659 | PGM1 | 1 | 0.05019 | 0.01399 | 0.4737 |
| rs5753037 | Type 1 diabetes | Other | ILMN_1679919 | ASCC2 | 22 | 0.6983 | 0.01686 | 0.93 |
| rs951005 | Rheumatoid arthritis | Other | ILMN_1664912 | IL11RA | 9 | 0.7907 | 0.03233 | 0.989 |
| rs1003719 | Eye color | Other | ILMN_1728605 | TTC3 | 21 | 0.3419 | 0.03324 | 0.4257 |
| rs5759167 | Prostate cancer | Other | ILMN_1663113 | TTLL12 | 22 | 0.6037 | 0.04204 | 0.7854 |
| rs1801133 | Plasma homocysteine | Blood | ILMN_1651385 | MFN2 | 1 | 0.4706 | 0.04299 | 0.8753 |

Footnotes

; This probe was annotated by hand to RPS26 on Chr12

^2^This probe was annoted by hand as NR_026680, a non-coding mRNA on Chr17
